# Not gently down the stream: flow induces amyloid bonding in environmental and pathological fungal biofilms

**DOI:** 10.1128/mbio.00203-25

**Published:** 2025-05-16

**Authors:** Peter N. Lipke

**Affiliations:** 1 Biology Department, Brooklyn College of the City University of New York, Brooklyn, New York, USA; Instituto Carlos Chagas, Curitiba, Brazil

**Keywords:** cross-β bond, adhesin, shear stress, catch bond, liquid flow

## Abstract

Surface-bound biofilms are the predominant microbial life form in the environment and host organisms. Many biofilms survive and thrive under physical stress from liquid flow in streams, fuel lines, blood, and airways. Strategies for biofilm persistence include shear-dependent adhesion (called catch bonding). In some cases, biofilms are physically strengthened by the formation of cross-β bonds between proteins: the same process that generates amyloids. Cross-β bonds have low dissociation rates. In biofilms, they bind cells to substrates, each other, and the biofilm matrix. Most fungal adhesins include amino acid sequences that can form amyloids. Shear flow activates these adhesins by unfolding pseudo-stable protein domains. The unfolding exposes sequence segments that can form cross-β bonds. These segments interact to form high-avidity adhesin patches on the cell surface. Thus, cross-β bonding is a consequence of flow-induced exposure of the cross-β core sequences. Liquid flow leads to both biofilm establishment through catch bonding and biofilm strengthening through amyloid-like bonds. This shear-dependent induction of biofilm establishment and persistence is a model for many microbial systems.

**IMPORTANCE:**

The microbes in biofilms persist in many environments, including industrial and pathological settings. These surface-associated communities show high resistance to antibiotics and microbicides. Biofilms also resist scouring by liquid flow. Amyloid-like cross-β bonds allow the establishment, strengthening, and persistence of many biofilms. This discovery opens a window on the novel use of anti-amyloid strategies to control microbes in biofilms.

## INTRODUCTION

Many biofilms form under flow and subsequently become highly shear resistant. In bacterial biofilms, shear resistance results from two mechanisms: adhesin catch bonds that strengthen under shear stress and stress-resistant fibers in the matrix. Many of these fibers are amyloids: cross-β-bonded aggregates of protein. In fungal biofilms, both catch bonding and flow resistance are mediated through similar cross-β bonding. Cross-β bonding of the adhesins makes high-avidity adhesive patches on the cell surface. Cross-β bonds also form between the cells to make strong amyloid-like adhesions.

Biofilms are microbial colonizations of surfaces, and they are ubiquitous (1). In the environment, they include the slimy layer on the surface of rocks in streams, as well as the lichens that decorate rocks worldwide, the “soap scum” and mildew in our showers, and fungal biofilms that clog fuel lines (2–4). Within ourselves, there are complex multi-species biofilms on mucus membranes and in the microbiomes on our skin and in our gut (5–8). Several diseases are classically biofilm-associated, including cholera, gingivitis, endocarditis, irritable bowel diseases, and cystic fibrosis (9). Indeed, it has been estimated that 60%–80% of systemic infections involve pathological biofilms (9, 10) (NIH PA-03-047). Biofilm characteristics include high drug resistance and great persistence (1, 11–15). These characteristics stem from the metabolic reprogramming. Among the consequences are altered nutritional metabolism and secretion of an embedding matrix of proteins, nucleic acids, and glycans that surround the cells (7, 8). Biofilms are often resistant to scouring at high pressure (16–18). So, biofilm-encrusted rocks are slippery even under waterfalls. Also, biofilms can foul industrial processes even after applications of antimicrobials and cleaning with high-pressure hoses. This characteristic bedevils food processing lines, leads to maritime fouling, and sometimes clogs fuel lines (19). As an example, *Pseudomonas fluorescens* grows well on a steel substrate, even at high shear (20). Furthermore, the biofilms grown at the highest shear are the most resistant to killing and removal by glutaraldehyde, benzalkonium chloride, or bleach. (In this study, the high shear stress was 4 Pa or 40 dynes·cm^−2^, equivalent to shear in the aorta.)

The effects of shear stress from liquid flow are the subject of extensive biofilm research (21–26). The persistence of biofilms is the result of catch bonding (bonds that strengthen under stress) and the viscoelastic properties of the matrix. These properties depend on the presence of fibrous and amorphous components of the matrices. In many cases, the fibers are composed of proteins that assemble into amyloids, a fibrous structure formed by cross-β bonding (27–30).

## CROSS-β BONDS AND AMYLOIDS

**Fig 1:**
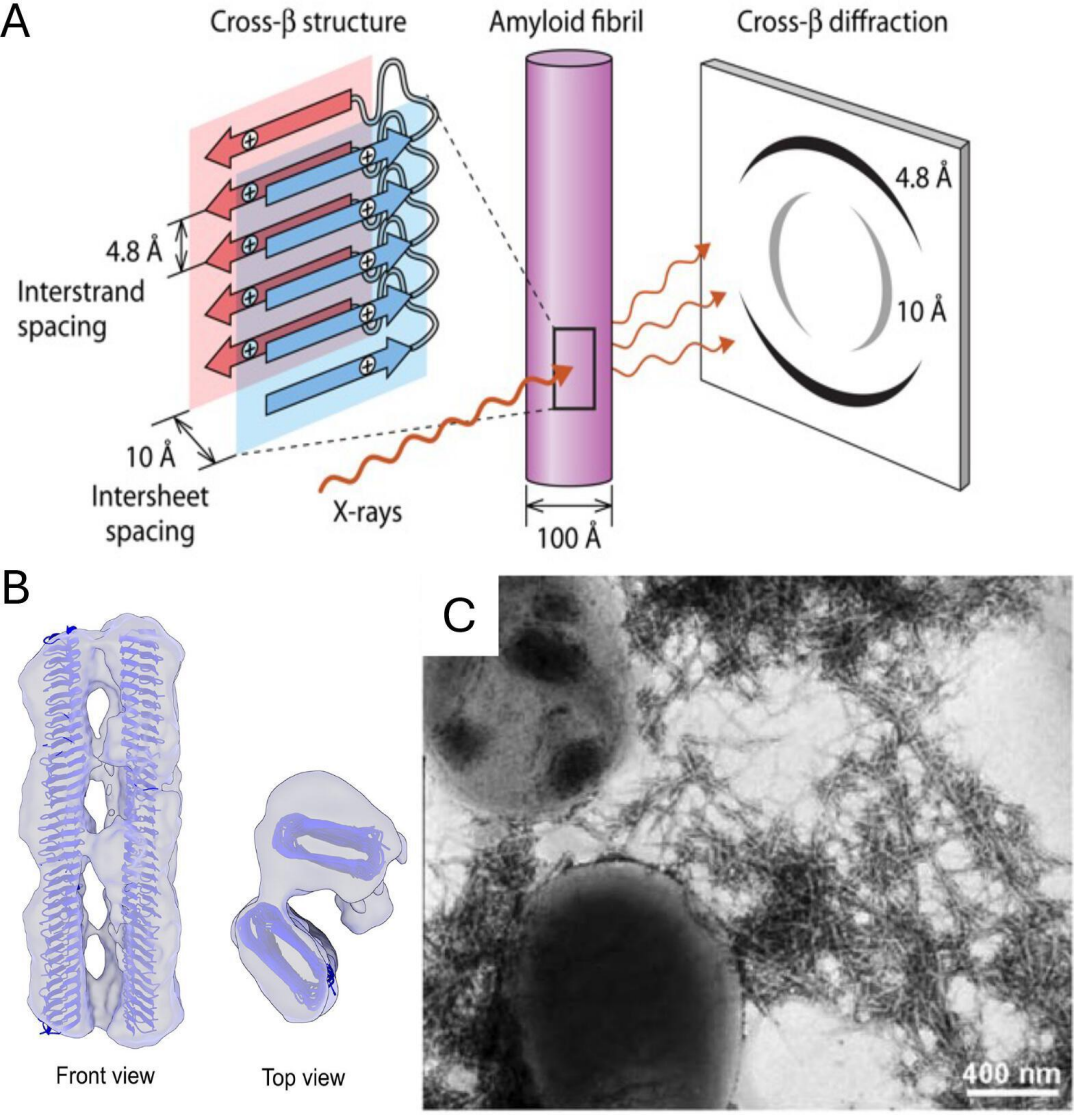
Amyloid structures and cross-β bonding. (A) Amyloid structure based on cross-β bonding to form fibrils with β strands perpendicular to the fiber axis. Blue and pink β-sheets are composed of β-strands, with identical or near-identical sequences. The blue sheet is composed of cross-β core sequences (adapted with permission from reference 24). (B) Cryo-EM model of two amyloid cross-β fibrils composed of *Escherichia coli* curli formed by CsgA (31). Reprinted with permission. (C) Electron micrograph of *E. coli* aggregation through curli (32). Reprinted with permission.

Some peptide sequences can form cross-β bonds with identical or highly similar sequences (27–30). These segments are often called “amyloid-forming regions” or “amyloid core sequences.” Here, I will refer to them as “cross-β core sequences,” a more general term. The amino acids in these cross-β core sequences form the β-strands in β-sheets composed of identical sequence segments (Fig. 1A). The β-sheets associate face-to-face or face-to-back, and the stack of sheets is stabilized by specific interactions of the amino acid side chains. At least some of these inter-sheet associations are hydrophobic and often anhydrous: i.e., there are regions between the sheets that contain no water (28). Such hydrophobic effect interactions stabilize aggregates under physiological conditions, because denaturants cannot gain access to the inter-sheet regions. In amyloid structures, the β sheets form fibers along their long axes, with the direction of the β strands perpendicular to the fiber long axis (Fig. 1). This arrangement generates the characteristic X-ray diffraction pattern with two major reflections at 90^o^ orientation; hence, the name “cross-β.” The β-sheet fibers, in turn, often bind to each other to form fibrous aggregates (Fig. 1). In amyloid disease states, the fibers aggregate, forming insoluble “amyloid plaques” that are markers of the diseases. In microbial functional amyloids, the cross-β bonding is similar, but the cross-β arrays form geometries different from canonical amyloid fibers (11, 28). The critical difference is that the proteins are bound to the cell surface, so some assembly patterns are not geometrically possible. Nevertheless, for these proteins also, the result is a stable array of identical protein molecules bound together through cross-β bonding.

Cross-β core sequence segments are typically five to seven amino residues long and are highly diverse. Surprisingly, such segments are present in about one-third of proteins (28, 29, 33). Almost always, these segments are buried inside well-folded protein domains, and so they are shielded from the solvent and cannot interact with each other. Only after the protein is unfolded do these segments become solvent-exposed and able to form cross-β structures. That is why many amyloids form only after proteins are denatured (28, 34, 35). In other proteins, the formation of cross-β bonds has been selected because an amyloid-like macromolecular structure is beneficial. The resulting aggregates are called “functional amyloids.”

## CROSS-β BONDS IN BIOFILMS

Archaeal, bacterial, and fungal biofilms all light up with amyloid stains, which bind to cross-β structures (11, 24, 36). The biofilms contain functional amyloids, which contribute to biofilm strength and persistence. We know some of the specific proteins and the interactions that are involved in these functions.

### Bacterial biofilms

Amyloids are key components of many bacterial biofilms. Among the best studied are curli expressed by many enterobacteria (4, 14, 31, 37–41). These adhesive pili assemble through cross-β bonding to form β-helical pili (Fig. 1). Curli are also shed into the biofilm matrix. They are inherently sticky, so they form assemblies of amyloid-like fibers within biofilm. Functional amyloid-forming proteins are also excreted by gram-positive bacteria, including Streptococci and Staphylococci (11, 21, 42–45). (*Bacillus subtilis* TasA protein forms a similar sticky fibrous matrix, but it does not have a cross-β structure [46].) The biogenesis and regulation of expression of these proteins are well-studied, and consequently, curli have been used as self-assembly platforms and in assays for anti-amyloid drug discovery (11, 14, 39, 42, 47, 48).

**Fig 2:**
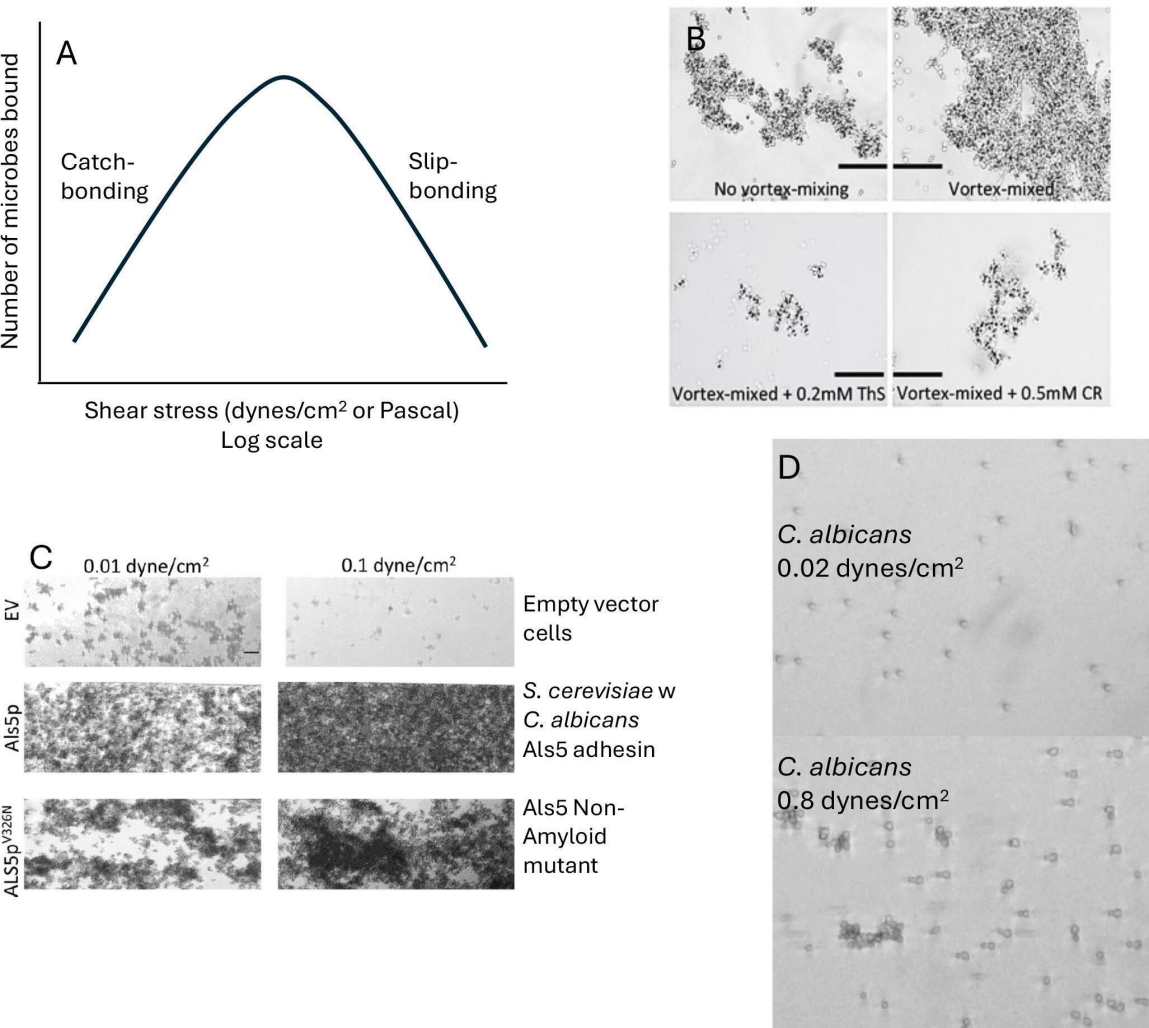
Effects of liquid flow on catch bonding and biofilm formation. (A) Catch bonding in microbial adhesins: adhesion increases as shear stress increases, typically from 0.01 to 10 dynes/cm^2^. The weakening of adhesion at very high shear is called slip bonding. Nevertheless, some biofilms persist on heart valves where shear stress can exceed 100 dynes/cm^2^ or 10 Pa. (B) Effects of vortex mixing on aggregation of *Saccharomyces cerevisiae* cells expressing *Candida albicans* Als5. Inhibition by amyloid dyes is also shown: ThS, thioflavin S; CR, Congo red. (C) Effect of shear stress on 24-hour biofilms of *S. cerevisiae* with or without the *C. albicans* adhesin Als5 (54). EV, empty vector. (D) Cross-β-dependent catch bonding in *Candida albicans*. The images are samples from micrographs of the surface of the flow chamber after 12 min of flow at the indicated shear stress. The videos are in Videos S1 and S2 in Chan and Lipke (54). Quantification of similar data has been published (54, 56, 57). Panels B and C are reprinted with permission (54).

In bacterial response to shear stress, a key event is catch bonding (Fig. 2) (16, 49, 50). Catch bonding is the ability of adhesins to bind more strongly under stress (forceful extension) than at rest: shear stress causes conformational changes in the adhesin. These changes result in a more extensive ligand-adhesin contact surface or a conformational change that blocks ligand exit from the binding site. Consequently, the dissociation rate becomes extremely slow or unmeasurable, and greater force is needed to disrupt the ligand-adhesin bond. Examples include lectins like FimH from gram-negative bacteria and mSCRAMMs from gram-positive bacteria (16, 49, 51, 52). These adhesins facilitate biofilm formation under stress by increasing adhesion to substrates. In some cases, they also increase bacterial aggregation in the early stages of biofilm formation (23, 53, 54). Of course, no bond can withstand infinite force, so enough shear will break any bond. The phenomenon of shear-dependent bond breaking is called slip bonding (Fig. 2). Catch bonding and slip bonding are typically observed as a flow-dependent binding of bacteria and fungi to ligand-coated surfaces (Fig. 2). Thus, there is a common model for response to flow and shear stress: catch bonding of adhesins leads to greater attachment to surfaces, and in some cases, to the capture of additional microbial cells by catch-bond-mediated aggregation. The biofilm then forms from the attached and aggregated cells (12, 19, 23, 55).

Biofilm formation itself often leads to the expression and excretion of functional amyloid proteins, with many of these proteins being expressed only as the cells enter the stationary phase (58). The model is that amyloids reinforce the matrix as biofilms mature. Mutants that do not express functional amyloid show weaker and less elastic biofilms, often by 2–3 orders of magnitude (11, 12, 48). Thus, amyloids stiffen the matrix and increase elasticity. Both catch bonding and biofilm functional amyloids contribute to persistence under shear stress and resistance to scouring by high-pressure fluid streams. Indeed, biofilm streamers induced under high flow are highly effective in clogging industrial processing lines, indwelling medical devices, and other high flow conditions (59). However, we do not know whether there is a role for functional amyloids in these phenomena. There is a recent extensive review on the effects of flow in bacterial biofilms (60).

Much work on the roles of amyloids in biofilms has been carried out in 96-well plates. However, the shear stress in these plates is not uniform: it is much higher for pellicles at the air-water interface than for bottom-attached biofilms (61). The latter are exposed to low shear even at moderate rotation speeds in orbital shakers. In most biofilm studies in microplates, rotation speed is not reported, but low shear speeds of 50–150 rpm are common.

### Functional amyloids in bacterial diseases

Fluid flow is a major factor in many diseases, and the best studied are in the pulmonary and gastrointestinal (GI) tracts. Flow is a major factor in *Pseudomonas aeruginosa* colonization of the airways in cystic fibrosis (62). The expression of the *P. aeruginosa* functional amyloid protein FAP increases adhesion and aggregation. It also strengthens the biofilms, increasing biofilm stiffness 20-fold (42). In a rat lung model, FAP expression is essential for airway colonization, immune escape, and metabolic reprogramming of the bacteria. Overexpression of FAP induces a CF-like biofilm state in control rats (63).

In the GI tract, the oral biome persists despite constant salivary flow and can become an origin for systemic infections. Biofilms of oral bacteria such as *Aggregatobacter, Fusobacterium*, and *Streptococcus* spp*.* stain with amyloid dyes, and colonization is inhibited by amyloid inhibitory compounds (43, 64–67). In *Streptococcus mutans,* adhesins Hsa, SerpA, and GspB are members of the MSCRAMM family. Like the fungal adhesins discussed below, they mediate catch bonding and have cross-β characteristics (21, 43, 68).

The commensal *Staphylococcus aureus* is a major cause of life-threatening systemic infections, and virulence is strongly associated with secretion of phenol-soluble modulins (9, 69). These peptides form unusual cross-α functional amyloids. In this amyloid structure, α-helices are arranged in parallel and perpendicular to the fiber axis, as if the β-strands in Fig. 1 were replaced by α-helices. Formation of these functional amyloids is strongly facilitated by shear stress (70).

In summary, functional amyloids and proteins that form cross-β structures are often associated with bacterial virulence and biofilm formation. In a few cases, there are reports that functional amyloid formation is facilitated by shear stress. However, more research is needed to clarify the relationships between shear, amyloid formation, and biofilm properties.

### Fungal biofilms

Different functional amyloids contribute greatly to the strength and persistence of mature fungal and bacterial biofilms (24). However, at least for yeasts, there is an additional function: cross-β bonds are also critical for catch bonding. Cross-β bonding is triggered by shear stress and leads to increased adhesion to surfaces and epithelia, as well as aggregation of the fungal cells in the initial stages of biofilm formation.

Yeasts also show catch-bonding behavior (Fig. 2). *Candida albicans* is an equal opportunity binder. It binds to most hydrophobic and biological substrates. Figure 2 and the associated videos show that binding to a BSA-coated surface is greater under 0.8 dynes/cm^2^ shear stress than at 0.02 dynes/cm^2^ (54). This behavior is replicated in a *Saccharomyces cerevisiae* model strain that expresses either *C. albicans* adhesin Als5 or Als1 (Fig. 2) (24, 71, 72).

**Fig 3:**
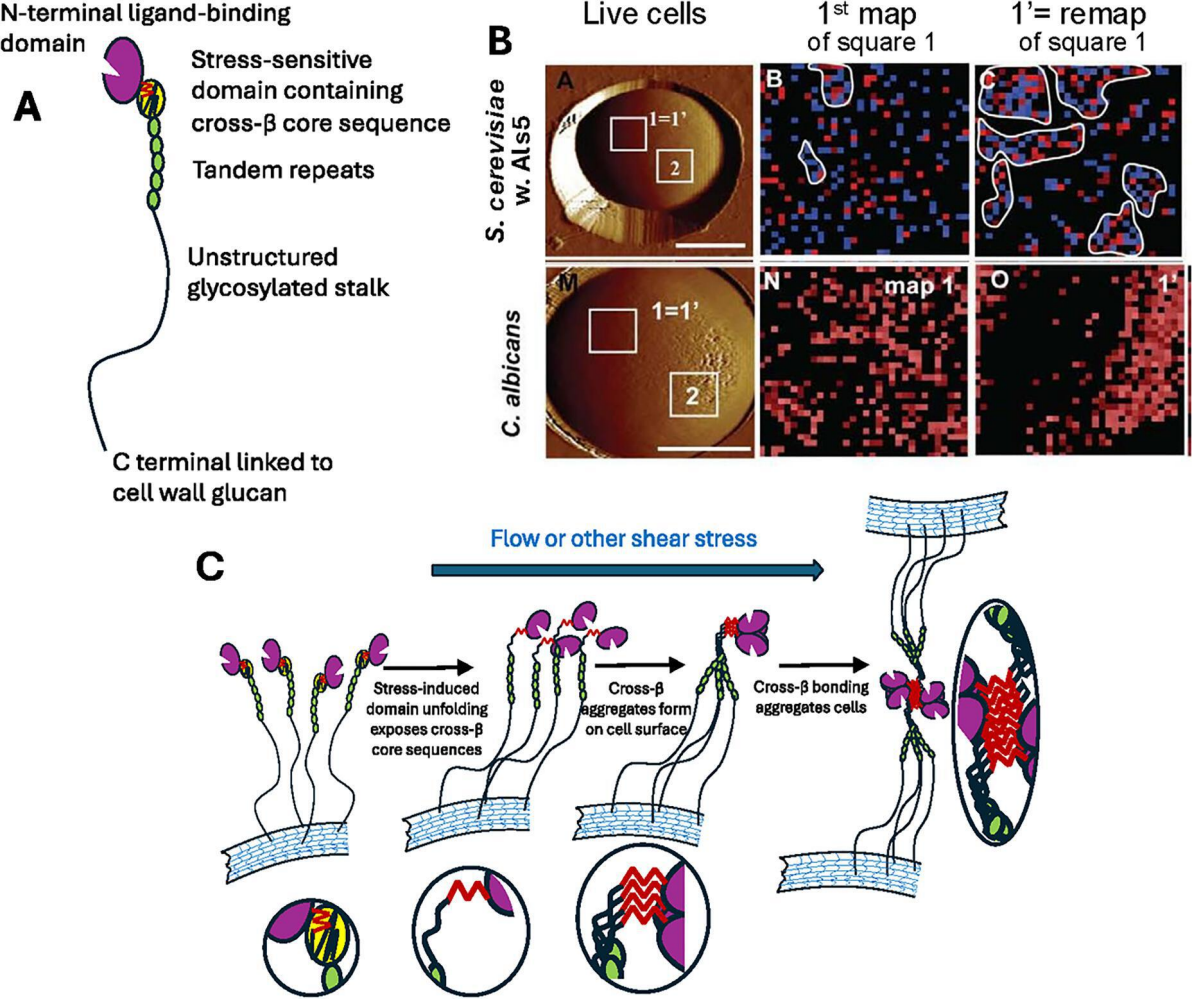
Cross-β dependent clustering of yeast adhesins. (A) Model of *C. albicans* adhesin Als5. Each adhesin has an N-terminal ligand-binding domain and a long, flexible disordered C-terminal stalk region. The stalks are covalently bound to cell wall polysaccharides, but their length and flexibility allow great mobility of the N-terminal domains. The cross-β core sequence is shown as a red zigzag in the yellow domain. (B) AFM mapping of surface adhesins (72, 78). The left image shows live cells as mapped, with 1 µm^2^ areas marked. The top row shows AFM mapping of the surface positions of *Candida albicans* Als5 adhesin expressed in a non-adhesive strain of baker’s yeast. Initially, the adhesins are randomly distributed in square 1. However, a second mapping of the same area (marked as 1’) shows the adhesins clustered together. The clusters propagate around the whole surface of the cell, increasing its adhesiveness (square 2, results not shown here). Red pixels show areas with rupture forces ≥150 pN; blue pixels have rupture forces between 20 and 150 pN. Areas outlined in white contain 10 or more contiguous colored pixels. The bottom row shows the same phenomenon for adhesins in *Candida albicans* itself. (C) A model for how the adhesins cluster: shear stress unfolds the yellow domain exposing the cross-β core sequence. The flexible stalks allow these sequences to form cross-β clusters on the cell surface. In the last step, two cells aggregate through cross-β bonding. The ovals show enlargements of the cross-β forming regions. Modified with permission (24, 72).

*C. albicans* biofilms are dependent on the expression of several adhesins, each with at least one strong cross-β core sequence. The cross-β bonds can form both *in vitro* and *in vivo* (24, 73–76). Figure 3A shows a model of an adhesin from *C. albicans*. These adhesins are fully active when expressed in non-adhesive lab strains of *S. cerevisiae* (24, 72, 77, 78). In the *S. cerevisiae* model system, atomic force microscopy (AFM) mapping shows that these adhesins are initially randomly distributed on the cell surface (Fig. 3B, middle column). Mapping itself administers stress to each mapped adhesin molecule as the AFM tip binds to the adhesin and then retracts away from the cell surface. Consequently, when the same area is mapped a second time, the adhesins cluster in patches on the surface of the cells (Fig. 3B, right column). The patches have characteristics of cross-β bonding (24, 72, 78, 79): mutation of the cross-β core sequence leads to a failure to form patches. Patches bind amyloid dyes at nanomolar concentrations, and their formation is inhibited at 1,000- to 10,000-fold higher concentrations (72).

**Fig 4:**
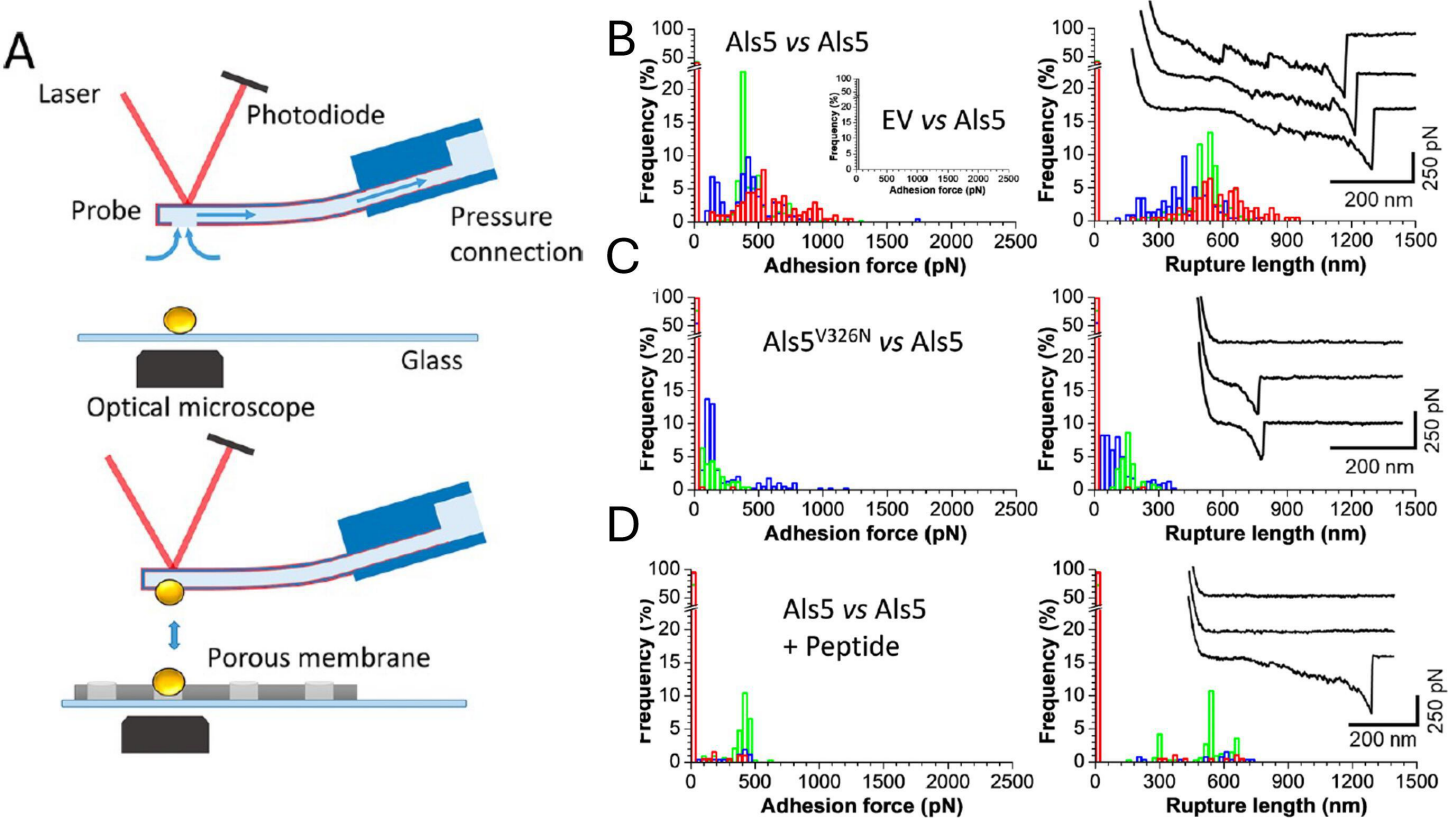
Single cell force microscopy (SCFM) of Als5-expressing cells. Reprinted with permission (80, 81). (A) SCFM procedure: a cell is suctioned onto a hollow AFM tip, then brought into contact with a cell on the microscope stage. The tip repeatedly contacts and withdraws from the surface, with force-distance analysis on each withdrawal. (B) Rupture force (left) and rupture distance (right) between cells expressing Als5. Each color represents a different pair of cells, with results of 1,000 contacts per pair summarized in the histogram. Inset shows a lack of adhesion when one cell does not express Als5. (C) Rupture forces and distances for pairs of cells with one cell expressing Als5 and the other expressing the non-amyloid Als5V326N mutant. (D) Summary of rupture forces and distances for pairs of Als5-expressing cells in the presence of a sequence-specific anti-amyloid peptide.

The cross-β bonding does not affect the binding domains immediately N-terminal to the cross-β core sequence. These domains retain their very broad ligand specificity and *K*_*D*_ values (72). Nevertheless, clustering of the adhesins increases the avidity of binding, because it increases the likelihood of multiple bonds being present at once; so clustering decreases the chance that all the bonds will be dissociated simultaneously. Bonds between clustered adhesins of tethered cells yield the thermodynamically lowest state because the strongest bonding becomes the lowest energy state (82). Therefore, clustering of adhesin molecules on the cell surface is a self-perpetuating physical state that traps other adhesins in the cross-β-bonded state, increasing the strength of the cell-cell bonds.

### How do shear-dependent cross-β bonds form?

When a fungal adhesin molecule is stretched in single-molecule AFM, the protein domain that contains the cross-β core sequence unfolds with very little force (Fig. 3C)(24, 83). Once this sequence is exposed to the solvent, it will associate with cross-β core sequences on nearby molecules to form cross-β bonds. The irreversibility of amyloid formation then assures that other molecules will add their cross-β core regions to the growing surface patch. The size of the cross-β bonded patches is limited by the number of adhesin molecules nearby, because the adhesins are covalently tied to the fibrous structural glucans of the cell wall. To facilitate the interactions, each adhesin molecule has a long, hyperglycosylated protein stalk that allows the N-terminal region, including the amyloid-forming sequence, to access a circular area with a diameter of 300–400 nm (0.12 µ^2^). Within this area, there are typically dozens to hundreds of adhesin molecules that can interact (78, 84). The result is that the adhesins are clustered into surface nanodomains of high avidity.

### The consequences of cross-β bond formation in fungal biofilms

Cross-β bond formation increases the number of cells in the biofilm by two- to threefold, even under low-shear conditions (24 hour biofilms in a 96-well plate rotated at 60 rpm [24, 72]). Because cross-β bonds form only after shear stress, they are catch bonds. This property is illustrated in Fig. 2 and the associated video, which show that *C. albicans* cells bind better to the surface of a laminar flow device when the shear stress is increased from 0.02 to 0.8 dynes/cm^2^, a 40-fold increase in force. Under the higher shear stress, the yeast cells also aggregate at a much higher level (Fig. 2) (24, 54, 56, 72, 85). The shear responses are accompanied by increased thioflavin fluorescence. The responses are abrogated in the presence of anti-amyloid compounds, including Congo red, thioflavins, and anti-amyloid peptides. The model yeast *S. cerevisiae* behaves similarly if it expresses cross-β-forming *C. albicans* adhesin Als5^WT^. The non-amyloid form Als5^V326N^ mediates neither thioflavin T (ThT) staining nor catch bonding. Also, biofilms subjected to moderate shear stress are much more resistant to being dislodged at high shear stress (20 dynes/cm^2^ or 2 Pa) (54). Again, the persistence is dependent on the ability of the adhesins to form cross-β bonds. Adhesins with these properties show behaviors that facilitate biofilm formation: adhesion to substrate, aggregation of the yeast cells, and resistance to dislodgement at high shear (24, 56).

Formation of biofilms in a laminar flow instrument is exquisitely flow-sensitive: biofilms were seeded with buffer flowing at a moderate rate, then developed for 12 hours in growth medium. At minimal flow, nascent biofilms develop (Fig. 2), but as the flow rate increases, the biofilms overgrow and block the flow channel (54, 85). In fact, the highest flow rate allowing 12-hour growth is only 0.1 dynes/cm^2^, 13% of the usual “moderate” shear in adhesion experiments. Shear stresses >0.1 dynes/cm^2^ invariably lead to blocked channels. Again, the shear-induced biofilm development is blocked by anti-amyloids or single-site mutation of the cross- core sequence in the adhesin. Thus, the bottom line is that shear stress activates the formation of cross-β bonding of fungal adhesins and leads to biofilms with greater mass and higher resistance to shear stress. These counterintuitive results are due to shear stress induction of cross-β amyloid-like bonds that cement biofilms together under flow.

Yeast cells also aggregate with each other by forming cross-β bonds between the cells (Fig. 3C and 4) (74, 80, 86). Single cell force microscopy (SCFM) measures the forces holding individual cells together. An intact cell is attached to the tip of an AFM with adhesive or by suction, then brought into contact with an immobilized cell on the microscope stage. As the tip is raised, the cells are pulled apart, and the force is measured at each separation distance (Fig. 4A) (86). There is cell-cell adhesion only if both cells express the adhesin. Figure 4B through D shows force-distance histograms of several pairs of cells, with the left histograms showing the frequency of ruptures at each shear force, and the right histograms showing the separation between the cells when rupture occurs. Four to seven times as many cell pairs bind to each other when both cells express cross-β-capable adhesins (Fig. 4B), relative to pairs with one cell being cross-β competent and the other expressing an adhesin mutant without a cross-β core sequence (Fig. 4C). Also, if both cells are cross-β competent, the mean strength of adhesion doubles. This activity for cross-β-forming adhesins is abrogated in the presence of amyloid inhibitors (compare Fig. 4B with Fig. 4D). The conclusion is that adhesins that form cross-β bonds greatly enhance the frequency and strength of cell-to-cell adhesion, and the results are consistent with the formation of cross-β bonds between cells.

The possibility of cross-β bonds in fungal adhesins can be predicted from protein sequence by algorithms designed to predict amyloid formation (28, 33, 83, 87, 88). Such predictors show widespread presence of amyloid-forming segments in known adhesins. The prevalence of such potential is high (>85% of fungal adhesins) (89). This frequency is much higher than in proteins in general. In the fungal adhesins, the cross-β sequences are often seen in pseudo-stable domains, as in the Als and Flo1-like adhesins.

We and others have demonstrated cross-β bonding in fungal adhesins from diverse gene families in *C. albicans* and non-albicans *Candida*. AFM experiments show similar amyloid patch patterns for *C. albicans* adhesins, including Als1 (74), Als5 (72, 78), and Pga59 (75), as well as Flo1 and Flo11 from *S. cerevisiae* (54, 77, 90). Similarly, there is evidence that cross-β bonding is involved in surface binding, aggregation, and biofilm formation in *C. albicans* (24, 83)*, Candida tropicalis* (91, 92), *Candida auris* (93), *Candida parapsilosis* (94, 95), and *Paracoccidioides* (96, 97). There are amyloid-like structures in biofilm abscesses in candidiasis, aspergillosis, coccidioidomycosis, histoplasmosis, and mucormycosis (98). The abscesses stain with thioflavin T and Congo red. They also bind the amyloid-specific innate immune receptor serum amyloid P component (SAP). SAP binding leads to skewing of macrophages to the non-inflammatory M2 subtype and secretion of anti-inflammatory cytokines (99). Consequently, there is a lack of inflammation and tolerance of fungal colonization (99–103). The conclusion is that cross-β bonding is extremely widespread in fungal biofilms.

## CONCLUSIONS

Biofilms are a natural state of microbes. Their persistence and drug resistance are problems in infectious disease and food and industrial processing as well (19, 104). Recent work shows that biofilms in our gut microbiome are normally tolerated but sometimes can contribute to chronic diseases (105, 106). Among the components of biofilms, functional amyloid proteins are major contributors to biofilm integrity, flow resistance, and response of the innate immune system.

Both bacterial and fungal biofilms form effectively under physical stress from liquid shear flow. Shear stress is also generated within tissues by host movement. Many microbes show catch bonding: they bind to surfaces under shear stress better than they bind in static conditions. The bound microbes become the seeds for mature biofilms. Consequently, catch-bond-seeded biofilms persist and often include cross-β-bonded amyloid fibers that resist scouring at high pressure. In fungal adhesins, liquid flow or other shear stress unfolds pseudo-stable protein domains. The unfolding exposes cross-β core sequences and leads to clustering of adhesins into high-avidity nanodomains on the cell surface. The high-avidity patches then form cross-β bonds between cells, forming aggregates that seed the biofilms. Thus, cross-β bonds both mediate catch bonding of fungal cells and increase biofilm cohesion and flow resistance. Thus, for bacteria, archaea, and fungi, amyloid-like bonding contributes to biofilm persistence under flow.

We have not yet exploited the discovery that cross-β bonding is a cause of biofilm persistence. In general, studies show that deletion of genes for functional amyloid proteins severely compromises biofilm formation (38, 40, 42, 64, 94, 107, 108). We fantasize that anti-amyloid treatments might successfully disrupt biofilms and make antimicrobials more effective. Such ideas have already been exploited in limited one-at-a-time assays: we have shown that anti-amyloids can disrupt *Candida* biofilms in a laminar flow device (54). Such assays could be used for more general screening in multichannel laminar flow devices, which could assay several compounds at once (21, 109, 110). Clearly, drug screening would require massively parallel laminar flow channels, each harboring a biofilm.

For instance, SDS is one of the few effective reagents for dissociating cross-β bonds; therefore, including it in our toothpaste is a good idea. However, preventing amyloid formation is currently much easier than disrupting existing amyloids. Thus, coating in-dwelling medical devices with anti-amyloids can prevent the formation of biofilms but is unlikely to dissociate pre-formed biofilms. Research into anti-amyloid prophylaxis may be productive for diseases with functional amyloids that have already been identified: cystic fibrosis (*P. aeruginosa*), oral thrush (*C. albicans*), or gingivitis (oral *Streptococcus*).

We now have some anti-amyloid treatments that have therapeutic potential as well. As an example, therapeutic monoclonal antibodies (mAbs) can disrupt Alzheimer’s disease amyloid plaques (111, 112). Indirect treatments also have benefits: in humans, serum amyloid P component (hSAP) is a soluble pattern recognition receptor present at high concentrations in serum. hSAP binds to amyloid deposits with high avidity and masks them from immune recognition (98, 99, 101, 103, 113–116). The drug miridesap dissociates hSAP from amyloids. A treatment combining miridesap with an anti-hSAP mAb clears amyloid deposits in serum amyloidosis patients (116). In mice, miridesap can prevent candidiasis-induced morbidity and mortality (115). Thus, we can imagine mAbs that would disrupt specific microbial functional amyloids; these would be used as adjuncts to antimicrobials for chronic infections. A fascinating idea is that miridesap might be useful in preventing Alzheimer’s disease (117, 118). Therefore, we need research into finding therapeutic approaches that mimic the phenotypes of mutations in microbial functional amyloid proteins. The field is wide open.

Box 1. **Glossary**

**Adhesin:** a microbial protein that mediates binding to substrate or other cells. Often, adhesins are covalently bonded to wall glycans.

**Affinity:** the microscopic binding constant, usually expressed as *K*_*D*_, the ligand concentration that leads to occupancy of 50% of the binding sites. Thus, smaller *K*_*D*_ values correspond to tighter binding.

**AFM:** atomic force microscopy.

**Amyloid:** fibrous protein aggregate formed through cross β-bonding of identical sequences in many protein molecules. Such aggregates are characteristic of many neurodegenerative diseases. The aggregates bind Congo red and thioflavins.

**Avidity:** the macroscopic binding constant. In this article, it refers to conditions required to dissociate whole cells. Avidity is typically proportional to the *K*_*D*_^*X*^, where *x* is proportional to the number of bonds formed between cells or between a cell and the substrate.

**Catch bond**: a bond that strengthens as shear or extension force becomes greater.

**Congo red:** a dye that stains cross-β and amyloid structures. High concentrations can inhibit amyloid formation.

**Cross-β bond:** a type of highly stable protein-protein interaction. The bonding includes β-sheets where each β-strand has an identical or near-identical sequence. The β-sheets then associate through close interdigitation of the amino acid side chains, with some of the sheet-sheet interactions being anhydrous.

**Cross-β core sequence** (also amyloid core sequence): a peptide sequence that can constitute all of the β-strands in a β-sheet. These sheets must also have the ability to mate with other β-sheets through sidechain interdigitation (see Fig. 1A and B). Cross-β core sequences are typically five to seven amino acids in length.

**Functional amyloid:** cross-β-bonded protein aggregate that is beneficial to the organism expressing the protein.

**Newton:** the SI unit of force. One newton is the force needed to accelerate a 1 kg mass by 1 m/s^2^. Typically, the force needed to separate adhered cells from the substrate or from each other is measured in piconewton to nanonewton (10^−12^–10^−9^ N). These quantities are measured by AFM in single-cell force microscopy and single-molecule force microscopy.

**Slip bond**: bond that dissociates as shear or extension force becomes greater.

**Shear stress:** the physical stress or pressure due to fluid flow, measured in dynes/cm^2^ or Pa (N/m^2^ = 1 kg/s^2^·m). 1 Pa = 10 dynes/cm^2^. Shear stress due to blood flow has been estimated at 5-300 dynes/cm^2^ = 0.5–30 Pa.

**Thioflavins:** fluorescent dyes that stain cross-β structures with high affinity. High concentrations can inhibit amyloid formation. Thioflavin S is a mixture of isomers, including thioflavin T.
